# Direct and Indirect Effects of Sex Steroids on Gonadotrope Cell Plasticity in the Teleost Fish Pituitary

**DOI:** 10.3389/fendo.2020.605068

**Published:** 2020-12-07

**Authors:** Romain Fontaine, Muhammad Rahmad Royan, Kristine von Krogh, Finn-Arne Weltzien, Dianne M. Baker

**Affiliations:** ^1^ Physiology Unit, Faculty of Veterinary Medicine, Norwegian University of Life Sciences, Oslo, Norway; ^2^ Department of Biological Sciences, University of Mary Washington, Fredericksburg, VA, United States

**Keywords:** estrogen, androgen, adenohypophysis, brain, gonads, plasticity, pituitary, steroids

## Abstract

The pituitary gland controls many important physiological processes in vertebrates, including growth, homeostasis, and reproduction. As in mammals, the teleost pituitary exhibits a high degree of plasticity. This plasticity permits changes in hormone production and secretion necessary to meet the fluctuating demands over the life of an animal. Pituitary plasticity is achieved at both cellular and population levels. At the cellular level, hormone synthesis and release can be regulated *via* changes in cell composition to modulate both sensitivity and response to different signals. At the cell population level, the number of cells producing a given hormone can change due to proliferation, differentiation of progenitor cells, or transdifferentiation of specific cell types. Gonadotropes, which play an important role in the control of reproduction, have been intensively investigated during the last decades and found to display plasticity. To ensure appropriate endocrine function, gonadotropes rely on external and internal signals integrated at the brain level or by the gonadotropes themselves. One important group of internal signals is the sex steroids, produced mainly by the gonadal steroidogenic cells. Sex steroids have been shown to exert complex effects on the teleost pituitary, with differential effects depending on the species investigated, physiological status or sex of the animal, and dose or method of administration. This review summarizes current knowledge of the effects of sex steroids (androgens and estrogens) on gonadotrope cell plasticity in teleost anterior pituitary, discriminating direct from indirect effects.

## Introduction

Teleost fish comprise the largest vertebrate group with close to 30,000 species (1), including well established model species such as zebrafish (*Danio rerio*) and Japanese medaka (*Oryzias latipes*), which provide valuable tools for basic research on vertebrate physiology (2, 3). In addition, numerous teleosts, such as salmonids, seabreams, basses, tilapia, and other species with regional importance, have commercial and ecological or societal value and are subjects of applied research related to aquaculture or conservation.

Compared to tetrapods, teleosts experienced an additional whole genome duplication, known as the 3R (4). Some teleosts, such as the salmonids, have also had a fourth duplication event (4R) (5). Therefore, teleost fish can potentially possess 2 to 4 times more genes than other vertebrates, and although many of the duplicated genes have been lost through teleost evolution, some have been conserved and developed new or expanded functions. Fish reproductive physiology has been extensively investigated over the last decades, due to the high economic interest of controlling fish reproduction in aquaculture species and for evolutionary aspects as the main regulatory mechanisms are conserved among vertebrates.

In all vertebrates, the reproductive function is controlled through the physiological connections of the brain - pituitary - gonadal (BPG) axis, where the pituitary gonadotropes play a central role (6, 7). Located in the anterior pituitary (adenohypophysis), gonadotropes produce and release into the blood circulation the two gonadotropins (follicle-stimulating and luteinizing hormones, Fsh and Lh, respectively) which stimulate gonadal gametogenesis and steroidogenesis. Gonadotropins are heterodimeric proteins consisting of an α-subunit, common to both Lh and Fsh, and a unique β-subunit that confers the biological specificity (8). Interestingly, contrary to mammals and birds, in teleosts, the two gonadotropins are generally produced by discrete gonadotrope cell types; Lh cells and Fsh cells (9, 10).

Located below the hypothalamus, the pituitary is composed of two main parts with different developmental origins. The neurohypophysis (posterior pituitary) originates from a down-growth of the diencephalon and contains projections from neuroendocrine cells mainly located in the preoptic-hypothalamic region of the brain. The anterior pituitary originates from the placodal ectoderm at the anterior neural ridge which invaginates and subsequently separates from the stomodeum, a thickening of the ectoderm that forms the epithelium of the oral cavity (11). The anterior pituitary contains several hormone producing cells, including the gonadotropes which are localized to the *proximal pars distalis* (PPD).

**Figure 1:**
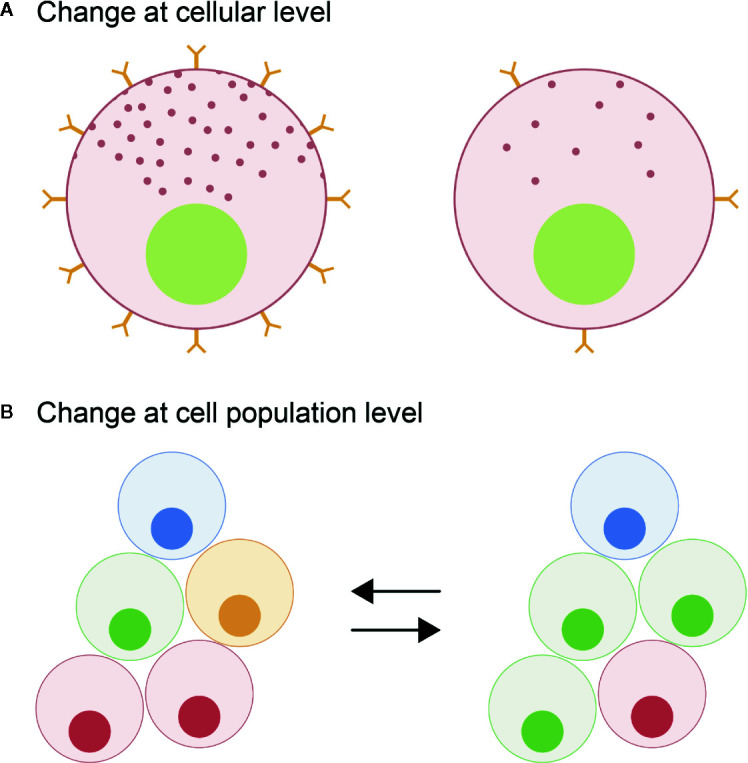
Schematic**** representation of the plasticity of the pituitary cells leading to a change in hormone production quantity. At the cellular level **(A)**, the activity of the endocrine cell (hormone synthesis and release) can be modulated through the regulation of the number of different receptors thus changing sensitivity of the pituitary cells to inputs and/or by changing the hormone production rates. At the population level **(B)**, the number of specific cell types can be modified changing the proportion of the different endocrine cell types in the pituitary.

**Figure 2:**
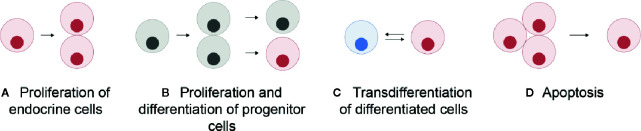
Schematic representation of the mechanisms allowing a change in the number of a specific endocrine cell type in the pituitary: proliferation (mitosis) of endocrine cells themselves **(A)**, proliferation of progenitor cells followed by their differentiation **(B)**, transdifferentiation (phenotypic conversion) of other differentiated cells **(C)**, and cell apoptosis **(D)**. Grey cells represent undifferentiated progenitor cells and colored cells represent differentiated cells.

Unlike in mammals where the different endocrine cell types are mosaically distributed in the adult anterior pituitary, in teleosts they are spatially discrete through the entire lifespan (6, 11). However, in both mammals and teleosts, the anterior pituitary shows high plasticity at both cellular and population levels, allowing the anterior pituitary to meet the demands for hormonal production as they change over the life cycle of an animal (12). At the cellular level, cellular activity (hormone production and release) can be modified by varying regulatory ligand sensitivity through the presence and number of receptors, or by altering rates of hormone synthesis and secretion, the latter corresponding to the hormone release as defined by Jena (13) (**Figure 1A**). At the population level, the number of cells of each endocrine cell type can change (**Figure 1B**). This can be due to proliferation of the endocrine cells (**Figure 2A**), differentiation of progenitor cells (**Figure 2B**), phenotypic conversion (transdifferentiation) of an endocrine cell into another cell type (**Figure 2C**), or cell death (apoptosis) (**Figure 2D**).

**Figure 3:**
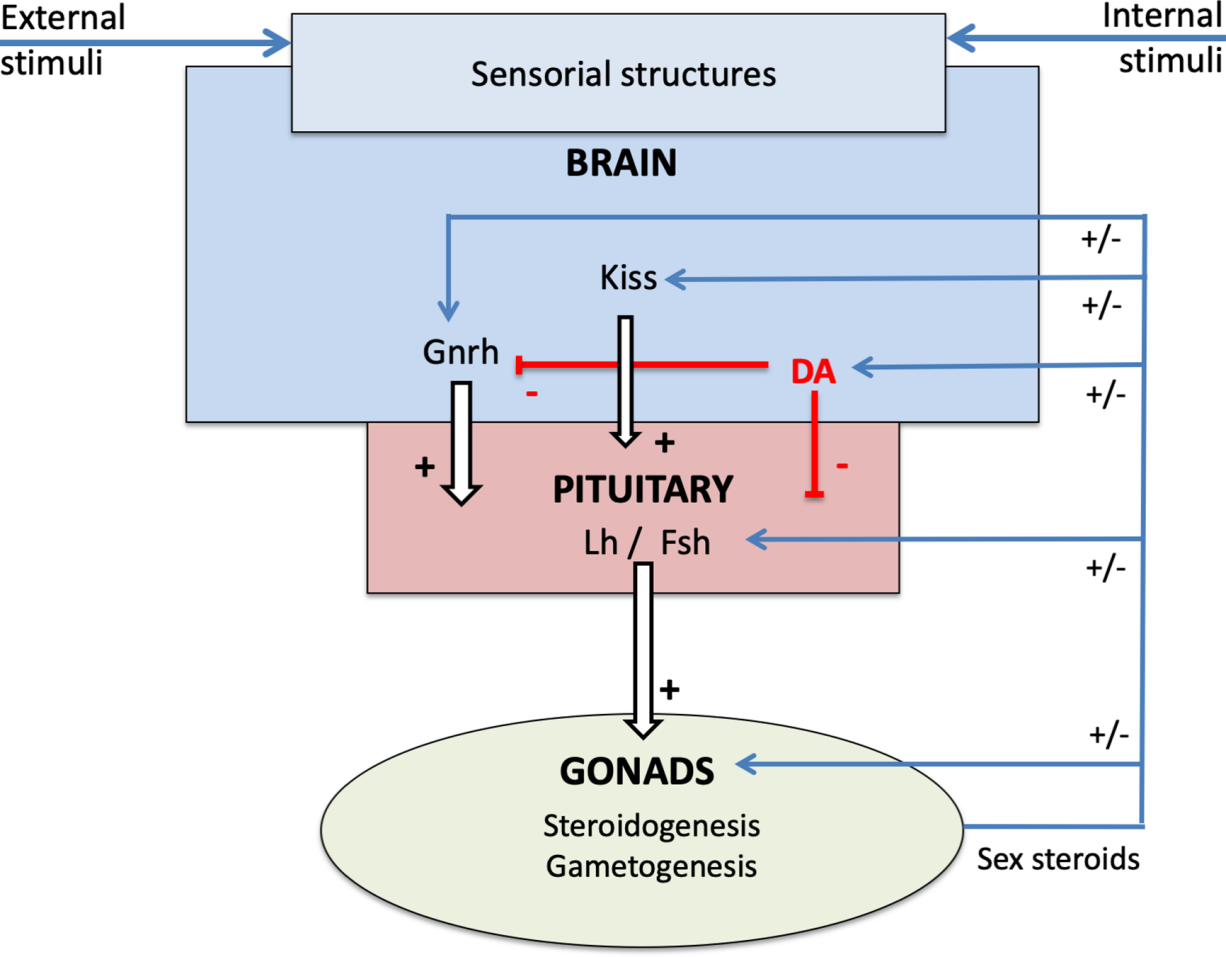
Schema of the neuroendocrine control of reproduction in teleosts, presenting the brain-pituitary-gonad (BPG) axis and the role of sex steroids in the retro-controls at the different levels.

The pituitary endocrine cell population with the highest capacity for plasticity is likely the gonadotropes. Gonadotrope plasticity (cell activity and cell number) is regulated by a myriad of brain factors primarily released from the preoptic-hypothalamic region. The main brain factors in this regard are gonadotropin-releasing hormone (Gnrh), dopamine (DA), and Kiss in teleosts (**Figure 3**; for review see (7, 14, 15)). Gnrh serves as the main stimulator of gonadotropes (7, 16). The neuro-hypothalamic Kiss system also plays an important stimulatory role (15). In contrast, DA appears to be the main gonadotrope inhibitor (7, 17). Other neuroendocrine factors regulating gonadotropes include Gnih, neuropeptide Y, GABA (7), but these will not be discussed in this review.

In addition to signals from the brain, the plasticity of gonadotropes is also modulated by negative and positive feedback *via* endocrine signals from peripheral organs. Among these signals are the sex steroids. Sex steroids are synthesized from cholesterol, predominantly by the steroidogenic cells of the gonads, and circulate at different levels in males and females (18). Two major classes of sex steroids, androgens and estrogens, were classically delineated as male- and female-specific hormones as they were mainly synthesized by the testes and ovaries, respectively, and found to promote male and female secondary sex characteristics. We now know that both androgens and estrogens are essential regulators in both males and females.

Steroidogenesis primarily occurs in the gonads, in testicular Leydig cells in males, and ovarian granulosa and theca cells in females (19). However, granulosa cells are not strictly steroidogenic but process steroid precursors from theca cells. For instance, testosterone (T) from theca cells is aromatized in granulosa cells to 17β-estradiol (E2), the most prevalent and potent form of circulating estrogen in fish, a process regulated through maturation-dependent levels of aromatase. Aromatase is a member of the P450 cytochrome enzyme superfamily and encoded by the *cyp19a1* gene, which exists in two forms in teleosts: *cyp19a1a* and *cyp19a1b*, the former expressed in the ovary and the latter in the brain and pituitary of both sexes (20, 21). While T and the even more potent, non-aromatizable, hormone 5α-dihydrotestosterone (DHT) are the active androgens in mammals, the non-aromatizable 11-ketotestosterone (11-KT) is the main active androgen in most teleosts (22, 23). However, DHT is also found in the circulation in both male and female fathead minnow (*Pimephales promelas*) (24, 25), and is the predominant steroid produced by urohaze-goby (*Glossogobius olivaceus*) testis tissue *in vitro* (26). Moreover, activity of 5-α reductase, which converts T to DHT, has been detected in many tissues, including the brain and pituitary, in several teleost species (25, 27), indicating that there may be a still undescribed biological role of DHT in teleosts. Finally, there is a third class of vertebrate sex steroids, the progestogens, which can be converted through several steps to T, 11-KT and E2, cortisol, and other steroids (18), but will not be further covered in this review.

Interestingly, in vertebrates, sex steroids can also be produced in other tissues, including the central nervous system, either *via* *de novo* synthesis from cholesterol or from other steroid intermediates produced in the periphery, thus allowing the tissue to autonomously utilize and modulate local steroid signaling (28, 29). In teleosts, expression of numerous enzymes, including aromatase, involved in sex steroid biosynthesis has been described in several brain areas (30, 31).

In target cells, sex steroids bind both nuclear and transmembrane receptors driving complex signaling responses (for reviews see (32–34)). In most vertebrates, genome duplications have led to two cytoplasmic estrogen receptors (ESR; ESR1 and ESR2), and one androgen receptor (AR). Teleost fish, however, often possess multiple paralogs of each receptor due to the additional whole genome duplications that occurred before and within the teleost group (3R/4R), adding complexity to sex steroid receptor signaling. Indeed, in most teleost species, while a single Esr1 has been maintained, two Esr2 (Esr2a and Esr2b), and two Ar (Arα and Arβ) have been conserved (35, 36).

Considering the progress in the field made in recent years, this review aims to summarize current knowledge regarding the effects of androgens and estrogens on gonadotrope activity and number in teleosts. Because these steroids can act on all levels of the BPG axis, we also aim to delineate their indirect effects *via* the brain from their direct effects on the gonadotropes.

## Sex Steroids Are Mediators of Gonadotrope Plasticity

### Effects on Gonadotrope Activity

#### Effects on Sensitivity to Regulatory Signals

Sex steroids can alter gonadotrope activity by changing gonadotrope cell sensitivity to regulatory signals, through modulation of the levels of Gnrh or DA receptors. Although molecular mechanisms by which steroids regulate Gnrh and DA receptors are well documented in mammals, much less is known in fish.

In mammals, GnRH signaling occurs through two paralogous receptors (37), whereas additional paralogs have been identified in teleosts, with up to six receptors in Atlantic salmon (38). In spite of this genomic difference, effects of sex steroids on GnRH receptor expression has been found across taxa. For example, E2 up-regulated pituitary *GnRH-R* expression in several mammalian studies, including in cows (39, 40), rats (41, 42), and ewes (43, 44), while T also up-regulated pituitary *GnRH-R* in rats (45). In teleosts, the effects of sex steroids on *gnrhr* expression have been investigated in only a limited number of species which have yielded disparate results. In the European sea bass (*Dicentrarchus labrax*), Gnrh receptor transcript levels in gonadotropes strongly increased at the time of spawning, when E2 is elevated (46), suggesting a stimulatory effect of E2 as seen in mammals. Conversely, in African catfish (*Clarias gariepinus*), castration increased pituitary Gnrh receptor content (47). This effect was reversed by treatment with androstenedione (AS), but not with non-aromatizable 11β-hydroxyandrostenedione (11β-OHA4). Finally, in goldfish (*Carassius auratus*), T-enhanced Lh-responsiveness to Gnrh was shown to be independent of changes in pituitary Gnrh receptor affinity or number (48).

Interestingly, among teleost species studied, only one of the multiple paralogs appears to be directly up-regulated by sex steroids in each species. Furthermore, if we apply the nomenclature and molecular phylogeny from (38) for *gnrhr* genes to the published studies, we find that it is the same isoform across species. For example, E2 treatment in female Nile tilapia (*Oreochromis niloticus*), increased mRNA levels of both *gnrhr2ba1* (*gnrhr1*, according to the authors) and *gnrhr2ba2* (*gnrhr3*) *in vivo*, but only *gnrhr2ba1* (*gnrhr1*) *in vitro*, suggesting E2 directly regulates *gnrhr2ba1* but indirectly regulates *gnrhr2ba2* (49). Similarly, in the male black porgy (*Acanthopagrus schlegeli*), E2 and T increased the expression of *gnrhr2ba1* (*gnrhrI*) (50, 51), but not *gnrhr1cb* (*gnrhrII*) (50). Finally, in pituitary cultures from Atlantic cod (*Gadus morhua*), E2 and T stimulated the expression of *gnrhr2ba1 (gnrhr2a)* but not *gnrhr1cb* (*gnrh1b)* in mature and post-spawning fish, and DHT increased expression of only *gnrhr2ba1* in post-spawning fish (52). While three species cannot represent the entire diversity of teleosts, they suggest that this receptor acquired a sensitivity to sex steroids in a common ancestor and this has been conserved through evolution.

In mammals, it is well established that E2 modulates pituitary expression of type 2 dopamine receptors (D2), as demonstrated in rats (53–55). Regarding DA receptors, two DA receptor families have been described in vertebrates: type 1 dopamine receptors (D1) and D2. Similar to Gnrh-r, multiple paralogs within each family are found in teleosts (56). *In vitro* experiments have demonstrated that pituitary D2, but not D1, plays a role in the dopaminergic inhibition of gonadotropin synthesis and secretion in several teleost species (17). D2 has been localized to the PPD in many teleosts, and to Lh cells in rainbow trout (*Oncorhynchus mykiss*) (57) and zebrafish (58). Sex steroid regulation of pituitary D2 has been studied in few teleost species, with divergent effects. In rainbow trout, D2 antagonist decreased the stimulatory effect of Gnrh3 on Lh cells (59), whereas in Nile tilapia, *d2* mRNA levels increased in females following E2 treatment, both *in vivo* and *in vitro* (49). In European eel, neither E2 nor T affected pituitary *d2* levels (60).

#### Effects on Hormone Synthesis and Secretion

Gonadectomy (GDX) experiments and *in vivo* or *ex vivo* steroid treatments in a wide variety of species have demonstrated the significant role of the gonadal feedback loop in regulating gonadotropin synthesis and secretion in teleosts, with sex steroids exerting both negative and positive effects (**Table 1**).

**Table 1:** Effects of gonadectomy or sex steroid treatments *in vivo* or *ex vivo* in different teleost species.

Species	Stages/sex	Pituitary synthesis		Secretion		Reference
		Lh	Fsh	Lh	Fsh	
African catfish (*Clarias gariepinus*)	immature M	GDX ↓ Lh T ↑ Lh (not 11-KT)				(61)
	mature M	GDX ↓ Lh E2 estrone T and AS, ↑ Lh (not DHT or 11β-OHA4)		GDX ↑; T and AS ↓ (not E2, E1, DHT or 11β-OHA4)		(62)
		GDX ↓ *lhb* 11-KT ↑ *lhb*				(63)
Atlantic croaker (*Micropogonias undulates*)	maturing mix M/F	E2 ↑ *lhb*	E2 ↓ *fshb*			(64)
	Late maturing/mature mix M/F	No effect of E2 or GDX on *lhb*	GDX ↑ *fshb* *E2* ↓ *fshb*	No effect of GDX or E2 on basal Lh level GDX ↑, and T and E2 ↓ Gnrh-induced stimulation	
	M			E2 ↑ (not T, DHT)		(65)
	immature M and F	T ↑ Lh				(66)
Atlantic salmon (*Salmo salar*)	maturing M	T and 11-KA ↑ Lh	Summer: GDX ↑ Fsh T and 11-KA ↓ Fsh		Summer: GDX ↑ T and 11-KA ↓	(67)
	mature M	GDX ↓ Lh T and 11-KA ↑ Lh	GDX ↓ Fsh 11-KA ↑ Fsh Summer: T ↓ Fsh Autumn: T ↑ Fsh	GDX ↓ T ↑ (not 11-KA)	GDX ↓ 11-KA ↑ Summer: T ↓ Autumn: T ↑	(68)
Black porgy (*Acanthopagrus schlegelii*)	immature M	E2 ↑ *lhb*	E2 ↑ *fshb*			(51)
	2 years old M			E2 ↑ (not T, 11-KT)		(69)
	mature M	E2 ↑ *lhb*		E2 ↑ (not T, 11-KT)		(70)
	M (spawning season)	E2 ↑ Lh (not T)		E2 ↑ (not T)		(71)
	protandric transition			E2 ↑		(72)
	M and F			E2 ↑ (not T, 11-KT)		(73)
Coho salmon (*Oncorhynchus kisutch*)	M and F	T and E2 ↑ *lhb* and Lh	E2 ↓ *fshb* in M (not in F) No effect of E2 or T on Fsh		T and E2 ↓	(74)
European eel (*Anguilla anguilla*)	silver (immature) F	E2 ↑ Lh (not T)		no effect of E2 or T		(75)
		T and E2 ↑ *lhb*				(76)
		E2 ↑ *lhb* (not T or DHT)	no effect of E2, T, or DHT on *fshb*			(77)
	silver M	T and E2 ↑ Lh				(75)
Goldfish *(Carassius auratus*)	immature mixed sex	T and 11-HA for 12 or 24h ↓ while 48,72 and 96h ↑ *lhb*				(78)
		E2 and T ↑ *lhb* (not 11-KT)	*E2, T and 11-KT* ↓ *fshb*			(79)
	early recrudescent M and F	11-KT ↓ *lhb* E2, and T in F, ↑ *lhb*	E2,T and 11-KT ↓ *fshb*	E2 ↑ in M only (not T or 11-KT)		(80)
	mature M and F	E2 ↑ *lhb* in M only (not T or 11-KT)	No effects of T, E2, or 11-KT on *fshb*	*T* ↑ in F only (not E2 or 11-KT)		(80)
	early recrudescent and mature F	no effect of GDX, T, E2, or 11-KT on lhb	*GDX* ↑ *fshb* *E2, T, and 11-KT* ↓ *fshb*			(79)
	mature F			E2 ↓		(81)
		no effect of GDX, T, or E2 on Lh		GDX ↑ T and E2 ↓		(82)
				no effect of E2 or T		(83)
	mature mixed sex	T and 11-HA ↑ *lhb*				(78)
	mature F + M (*in vivo* and *ex-vivo*)	E2 ↑ *lhb* and Lh	E2 ↑ *fshb*			(84)
Hybrid striped bass	female (mid-vitellogenesis)	no effect of GDX on Lh GDX ↑ *lhb* E2 ↓ *lhb* and Lh	GDX ↑ *fshb* E2 ↓ *fshb*			(85)
Indian catfish (*Heteropneustes fossilis*)	F (preparatory phase)	GDX ↓ lhb E2 ↑ lhb	GDX ↑ *fshb* E2 ↓ *fshb*			(86)
	F (resting phase)	no effect of GDX or E2 on *lhb*	GDX ↑ *fshb* E2 ↓ *fshb*			
	mature F			GDX ↑		(87)
Japanese eel (*Anguilla japonica*)	silver	E2 ↑ Lh		E2 ↑		(88)
Masu salmon (*Oncorhynchus masou*)	yearling and 2 years old	no effect of GDX on Lh MT ↑ Lh		GDX ↑		(89)
Medaka (*Oryzias latipes*)	mature F	E2 and 11KT ↑ *lhb*	E2 and 11KT ↓ *fshb*			(9)
	mature F and M	E2 ↓ *lhb* in F no effect of E2 on *lhb* in M				(90)
	mature M and F	E2 ↓ *lhb*				(91)
Nile tilapia (*Oreochromis niloticus*)	F (early vitellogenesis)	E2 ↓ *lhb*	E2 ↓ *fshb*	E2 ↓	No effect of E2	(49)
Orange‐spotted grouper (*Epinephelus coioides*)	mature F	no effect of GDX or E2 on *lhb*	GDX ↑ *fshb* E2 ↓ *fshb*			(92)
Ricefield eel (*Monopterus albus*)	*in vitro* pituitary fragments from F	E2, T and 11-KT ↑ *lhb*	no effect of E2, T, or 11-KT on *fshb*			(21)
Rainbow trout (*Oncorhynchus mykiss)*	F (early vitellogenesis)	E2 ↑ Lh		no effect of E2		(93)
	mature M (spermiation)			GDX ↑ E2, T, and 11-KT ↓		(94)
	mature M (early spermatogenesis)			GDX ↑ E2 and T ↓	
	mature M (resting phase)			GDX and T ↑	
	mature M (late spermatogenesis)			No effect of GDX, T, or E2		(94)
	immature M and F	T ↑ *lhb* and Lh		T ↑ Gnrh-induced stimulation		(66, 95, 96)
	mature F (late vitellogenesis)			GDX ↑ no effect of E2		(97)
	mature F (post ovulatory)			no effect of GDX or E2	
	mature F (germinal vesicle migration)			GDX ↑ E2 ↓	
	Immature M and F	T only in F, and E1, E2, and others* ↑ Lh				(98)
	organ culture from immature F	MT and E2 ↑ Lh		no effect of MT or E2		(99)
	immature M			T ↑		(100)
	immature F	E2 ↑ Lh		No effect of E2		(101)
	triploid (immature) F	T and E2 ↑ Lh		T ↑ (not E2)		(102)
	immature yearlings	T and E2 ↑ Lh	T ↑ Fsh E2 ↓ Fsh	T↑ (not E2)	T ↑ (but not E2)	(103)
	vitellogenic F			No effect of GDX or E2	GDX ↑ E2 ↓	(104)
	immature F			No effect of E2	E2 ↓
	mature F				T (and E2) ↓	(105)
	previtellogenic F			E2 ↑	E2 ↓ Fsh	(57)
	F			No effect of GDX or E2	GDX ↑ E2 ↓	(106)
Red sea bream (*Pagrus major)*	immature M	T ↓ *lhb* (not E2 or 11-KT)	11-KT and T ↓ *fshb* (not E2)	T ↓ (not E2 or 11-KT)		(107)
	mature M	11-KT ↑ *lhb* (not E2 or T)	No effect of E2, T, or 11-KT on *fshb*	No effect of E2, T or 11-KT		
Sablefish (*Anoplopoma fimbria*)	prepubertal F	E2 and T ↑ *lhb*	no effect of E2 or T on *fshb*			(108, 109)
Sea bass (*Dicentrarchus labrax*)	mature M and F	E2 T or DHT ↑ Lh; T ↑ *lhb* (not DHT or E2)	E2, T or DHT ↓ *fshb*	no effect of E2, T or DHT		(110)
	mature F and M	no effect of GDX or T on *lhb*	(GDX ↑ *fshb*) T ↓ *fshb*			(111)
Striped bass (*Morone saxatilis*)	immature F	T ↑ Lh		no effect of T		(112)

M, Males; F, Females; 11β-OHA4, 11β-Hydroxyandrostenedione; 11-HA, 11-hydroxyandrosterone; AS, androstenedione; E1, Estrone; 11-KA, 11-ketoandrostenedione; Others* include estriol, MT, testosterone propionate [11-oxygenated steroids, 11-KT, and 11-HA]; []: high concentrations are needed to provide significant differences.

Clearly, the effects of sex steroids on Lh and Fsh synthesis depend on developmental stage, reproductive status, sex, and even the duration and dose of the experimental treatment. For instance, in goldfish, *lhb* mRNA levels increased in juveniles, but not adults, following T and E2 treatments (79, 82). Sex-specific effects have been seen in European eel (*Anguilla anguilla*), where intraperitoneal E2 injections strongly increased pituitary Lh levels in immature eels of both sexes, while T strongly stimulated pituitary Lh level only in males (75). Similar sex-specific effects were observed for *fshb* transcripts in coho salmon (*Oncorhynchus kisutch*), where E2 was found to inhibit *fshb* in males but not in females (74). Dose-specific responses were reported in medaka, where high concentrations of E2 decreased *lhb* mRNA levels (90, 91) while more physiological levels stimulated *lhb* synthesis (9). Interestingly, in coho salmon, pituitary Fsh levels increased following administration of the non-aromatizable androgen 11-ketoandrostenedione (11-KA, a precursor of 11-KT), whereas T suppressed pituitary Fsh in summer but stimulated it in autumn (113), suggesting season-specific functions of T and an important role of aromatase in mediating negative feedback.

Secretion of Lh and Fsh are also affected by sex steroids, with differential effects depending on the species, sex, and/or maturational stage of the animal (**Table 1**). For instance, in goldfish, Lh release was stimulated by T in mature females and by E2 in early recrudescence in males (80). Studies in rainbow trout reported that T increased Fsh plasma levels in immature fish (103) but decreased in mature fish (104–106). Opposite effects were found in mature male Atlantic salmon (*Salmo salar*), in which T inhibited Fsh release in summer during gonadal maturation, but stimulated Fsh release during the autumn spawning period (68). Sex steroids can also influence gonadotrope cell activity by modulating gonadotrope response to Gnrh. For instance, in Atlantic croaker (*Micropogonias undulates*), a study found no effect of E2 on basal plasma Lh levels but an inhibitory effect on Gnrh-induced Lh release in mixed-sex adults (64). In goldfish, E2 and T potentiated the Gnrh agonist (Gnrh-a) effect on Lh secretion *in vivo* in a season-dependent manner (83)

Gonadotropin synthesis and release can be differentially affected by sex steroids within the same organism, as shown for example in rainbow trout where E2 stimulated Lh synthesis but did not affect Lh release (93, 99, 101–103). Finally, Lh and Fsh can be oppositely regulated within in the same species, both at the secretion and expression levels. For instance, in previtellogenic female rainbow trout, E2 implants increased plasma Lh level but decreased plasma Fsh (57), and in male coho salmon, E2 increased pituitary mRNA levels of *lhb* but decreased *fshb* (74).

### Effects on Gonadotrope Cell Populations

In addition to changes in gonadotrope cell activity, plasticity also results from changes in gonadotrope numbers and pituitary reorganization (**Figure 2**). For example, in European sea bass, while gonadotropes are only located in the PPD in immature fish, they tend to also colonize the periphery of the *pars intermedia* (PI) during maturation (46). Such population-level plasticity can make it difficult to discern whether steroid-induced changes in hormone mRNA or protein levels, detected by quantitative approaches such as ELISA or qPCR on the whole tissue, are due to changes in cell activity or cell number. Gonadotrope population changes in the pituitary can be due to proliferation of gonadotropes (**Figure 2A**), differentiation of progenitor cells, (**Figure 2B**) transdifferentiation (**Figure 2C**), and cell death (**Figure 2D**). These mechanisms underlie many changes in the rates of synthesis and release of Fsh and Lh by the pituitary, and there is increasing evidence that the sex steroids may play a role in these processes.

#### Proliferation

In teleosts, the pituitary grows throughout the lifespan. Evidence that pituitary endocrine cells are mitotically active has been illustrated at the electron microscope level in the mosquito fish (*Gambusia affinis*) (114). More specifically, the number of gonadotropes can change according to life stage or other factors, such as social status. For example, Lh cell number increased in juvenile male African catfish as spermatogenesis progressed (61, 115), and in both male and female medaka, the numbers of both Lh and Fsh cells increased between juvenile and adult stages (91, 116). In Nile tilapia, Fsh cell numbers were higher in dominant than subordinate males (117).

Furthermore, some studies demonstrate that gonadotrope proliferation can be controlled by sex steroids. In medaka, for instance, gonadotrope proliferation following steroid treatment was documented using double staining with proliferation markers Pcna and BrdU (91, 116). Exposure to T or E2, but not 11-KT, stimulated Lh and Fsh cell proliferation in both males and females, suggesting a positive effect of E2 on gonadotrope cell proliferation. In contrast, in zebrafish larvae, the number of Fsh cells was significantly lower in E2-treated fish than in controls (118), suggesting that E2 inhibits Fsh cell proliferation during early development in zebrafish. An earlier study in juvenile male African catfish showed that the number of Lh gonadotropes with numerous Lh-containing granules increased following T treatment (61). However, the authors did not detect differences in pituitary cell proliferation between androgen-treated and control fish and therefore speculated that androgens might activate quiescent gonadotropes. Whether these divergent results are due to species or stage differences remains to be investigated.

#### Differentiation of Progenitor Cells

Differentiation of progenitor cells may also increase gonadotrope cell numbers, but the evidence of a role of steroids in this process is not conclusive. In mammals, *Sox2*-expressing cells were shown to comprise a pool of pluripotent progenitor cells that proliferate and differentiate to either replenish pituitary cell populations or increase the absolute numbers of cells, including gonadotropes (119–122). These multipotent progenitor cells have been found in the pituitary cleft of mice (123–125) and rats (125, 126), lining the intraglandular structure bordering the adenohypophysis and neurohypophysis. While E2 treatment inhibited *SOX2* expression in human embryonic stem cells *in vitro* (127), other studies suggest that SOX2^+^ cell proliferation is stimulated by E2 treatment (128). This indicates that progenitor stem cell proliferation, and perhaps differentiation, may be regulated by sex steroids in mammals. In teleosts, however, multipotent *sox2*-expressing cells have only been detected in the brain (129–132) and retina (133). Pituitary *sox2*-immunoreactive cells have, to our knowledge, only been identified in one teleost study, where they were localized at the junction of the adenohypophysis and neurohypophysis in medaka (91). However, evidence that these cells are pluripotent and contribute to endocrine tissue renewal in teleosts is lacking.

Another marker, S-100, has been widely used to identify follicular stellate (FS) cells, which are non-endocrine cells networked by gap junctions throughout the anterior pituitary in mammals [for review, see (134, 135)]. FS cells are thought to be progenitor cells and thus involved in pituitary cell renewal and plasticity (136, 137). Interestingly, mammalian FS cells have been found to be sex steroid sensitive. Indeed, in male rats, GDX decreased the number of gap junctions, but T replacement maintained their numbers (138, 139). Similar observations were made in females where OVX reduced the gap junction number while E2, and to a lesser extent T, partly restored it (140). However, the role of sex steroids in FS cell proliferation or differentiation remains unknown in mammals.

Pituitary non-secretory (agranular) cells have been described in several teleost species, including the southern mouth-brooder (*Pseudocrenilabrus philander*) (141), sailfin molly (*Poecilia latipinna*) (142), ironfish (hybrid between the Funa (*Carassius carassius*) and goldfish) (143), stickleback (*Pungitius pungitius L*) (144), European eel (145), Mediterranean yellowtail (*Seriola dumerilii*) (146), grey mullet (*Mugil cephalus*) (147), Arabian toothcarp (*Aphanius dispar*) (148), and white seabream (*Diplodus sargus*) (149). In Nile tilapia, FS cells were observed to network *via* gap junctions (150). In the Japanese eel (*Anguilla japonica*), it was demonstrated that aromatase-positive cells, most likely corresponding to FS cells, express the proliferation marker PCNA (151). Such cells are suspected to have the same origin as the aromatase positive radial glial cells in the brain that act as progenitors throughout life (152). These results suggest that the proliferation of such cells could be sex steroid dependent, but this remains to be systematically investigated.

#### Transdifferentiation

The third mechanism that remodels gonadotrope populations is known as transdifferentiation, defined by (153–155) as the change from one hormone producing cell type into another. Transdifferentiation may allow the pituitary to appropriately respond to certain physiological and pathological conditions (156). While experiments have generated evidence of the phenomenon, the molecular mechanisms mediating such transformations are still enigmatic.

In teleosts, a recent study in medaka demonstrated that Fsh cells commenced *lhb* production *in vitro*, indicating the capability of a fully differentiated cell to transdifferentiate into another cell type (116). However, transdifferentiation between other pituitary cell types has not been reported in teleosts, and the role of sex steroids in transdifferentiation has not been investigated to date.

In contrast, several examples of pituitary endocrine cell transdifferentiation have been described in mammals. For instance, a study in adult mice found that stem-somatotropes can populate the pituitary with both somatotropes and lactotropes (157, 158). Similarly, studies in rats (159, 160) and humans (161) suggest that the proportions of somatotropes, lactotropes and mammosomatotropes (GH^+^/PRL^+^) in the adenohypophysis vary among non-pregnant, pregnant and lactating females due to cell transdifferentiation. More direct evidence of transdifferentiation of somatotropes into lactotropes is provided by *in vitro* studies (162, 163). In mammalian species, reversible interconversion has also been observed between somatotropes and lactotropes (164), somatotropes and thyrotropes (130, 153, 165–167), and somatotropes and gonadotropes (168).

While direct evidence for the role of sex steroids in cell phenotypic interconversion is also limited in mammals, there is some indirect evidence. In female dogs, estrogen deficiency due to ovarian dysfunction leads to an increase in gonadotropes, attributed in part to transdifferentiation (169). In rats, the production of *Fshb* and *Lhb* by somatotropes was coincident with a dramatic increase in *Esrβ* expression (168), suggesting that estrogen may regulate gonadotrope population remodeling *via* transdifferentiation.

#### Cell Death

Pituitary cell apoptosis is considered necessary to ensure the balance between cell renewal and cell loss and permit optimal response to physiological demands (170). Although experimental evidence of sex steroids regulating endocrine pituitary cell apoptosis is lacking in teleosts, there is ample evidence in mammals of both androgens and estrogens modulating apoptosis in such cells, including gonadotropes (170, 171). For instance, in proestrus rats, E2 has been reported to increase anterior pituitary cell apoptosis, predominately in gonadotropes, both *in vitro* and *in vivo* (172, 173). Another study in female rats reported that gonadotrope proliferation is lowest during diestrus (when E2 is lowest) before rising gradually until the estrus phase (174), suggesting that E2 may exert either anti-proliferative or apoptotic action to maintain an appropriate gonadotrope population in mammals. Whether apoptosis regulates the number of endocrine cells in teleosts remains to be elucidated.

### Conclusion

As shown above, it is now well established that sex steroids participate in the regulation of gonadotrope plasticity. The effects mainly occur at the cellular level by modulating the sensitivity of the endocrine cells to ligands through the regulation of the number of receptors, or by regulating endocrine cell activity (hormone synthesis and release). It also appears that in at least some species, increased gonadotropin production can result from gonadotrope cell division or progenitor cell differentiation. However, these and other population-level processes remain poorly characterized in teleosts. Therefore, there is a need for further studies to elucidate the underlying mechanisms regulating gonadotrope cell number and the potential role of sex steroids.

## Sex Steroids Mediate Gonadotrope Plasticity Directly and *via* the Brain

Because of the strong regulation of gonadotropes by the brain, it can be difficult to distinguish whether signaling molecules act directly at the pituitary, or instead modulate the gonadotrope regulatory systems in the brain. For example, in *ex vivo* medaka brain and pituitary preparations, Fsh cells exhibited a calcium response to Gnrh stimulation (175), but in dissociated cell cultures Fsh cells did not (176), suggesting an indirect effect of Gnrh on Fsh cells. For sex steroids, it is particulary complicated as sex steroids can simultaneously act at all levels of the BPG axis, making it difficult to determine whether the observed effects are directly on gonadotropes, mediated through the brain or another pituitary cell type, or both. Therefore, a combination of *in vivo* or *ex vivo* and *in vitro* techniques can help discriminate direct from indirect effects. In addition, investigation of Esr and Ar expression in brain and pituitary cells *in vivo* have identified some direct targets of sex steroids and thus have helped to decipher the pathways used for the regulation of gonadotrope plasticity, although much work remains.

### Brain Mediated Effects

ESRs, and to a lesser extent ARs, have been found to be widely expressed in the brains of vertebrates (177, 178), including teleosts (**Table 2**). While *esr*s and *ar*s are expressed in many different brain areas, numerous teleost studies have demonstrated that both *esr*s and *ar*s are highly expressed in the classical neuroendocrine regions of the brain such as the POA and the mediobasal and caudal hypothalamus (221). The high expression of *esr*s and *ar*s in the brain provides histological support for the sex steroid feedback control on gonadotropin synthesis/secretion and gonadotrope proliferation which may be relayed through regulators from the brain, notably Gnrh, DA, and kisspeptins. Therefore, any change in neuronal activity (synthesis or secretion) or cell number (neurogenesis or neurodegeneration) in the populations producing such factors might affect gonadotrope activity.

**Table 2:** Literature on the expression of androgen and estrogen receptors in the brain and the pituitary of teleosts.

Species	PITUITARY					BRAIN				
	ARα	ARβ	ESR1	ESR2a	ESR2b	ARα	ARβ	ESR1	ESR2a	ESR2b
African cichlid (*Astatotilapia burtoni*)	(179, 180)		(180)			(179, 180)		(180)		
Atlantic croaker (*Micropogonias undulates*)								(181, 182)		
Eelpout (*Zoarces viviparus*)								(183)		
European eel (*Anguilla anguilla*)	(184)									
Fathead minnow (*Pimephales promelas*)			(185)					(185)		
Goldfish (*Carassius auratus*)	*(186) (??)*		(187–189)			*(186) (??)*		*(190) (??)* (189, 191)		
Medaka (*Oryzias latipes*)	(192)		(91)					(193)		
						(194, 195)				
Midshipman (*Porichthys notatus*)			(196)			GENE LOST	(197)	(196, 198)		
Orange‐spotted grouper (*Epinephelus coioides*)								(92)		
Oyster toadfish (*Opsanus tau*)			*(199) (??)*					*(199) (??)*		
Paradise fish (*Macropodus opercularis*)			*(200) (??)*					*(200) (??)*		
Pejerrey (*Odontesthes bonariensis*)								(201)		
Platyfish (*Xiphophorus maculatus*)								*(202) (??)*		
Rainbow trout (*Oncorhynchus mykiss*)			(203–205)	(205)				(204, 206–210)		
Ricefield eel (*Monopterus albus*)			(211)	(212)					(212)	
Sablefish (*Anoplopoma fimbria*)	(213)		(213)			(213)		(195, 214)		
Sea bass (*Dicentrarchus labrax*)			(215)	(216)				(215)	(216)	
								(217)		
Sea bream (*Sparus auratus*)			(218)							
Zebrafish (*Danio rerio*)			(219)			GENE LOST	(220)	(219)		

Italic references followed by (??) show identification of receptor without subtype specificity.

#### Gnrh System

The Gnrh system is the main stimulator of gonadotrope cell activity. Gnrh exerts its effects through three Gnrh paralogs, classified according to lineage: Gnrh1, Gnrh2, and Gnrh3 (222, 223). While both Gnrh receptors and Gnrh3 or Gnrh2 fibers have been observed in the retina and the pineal gland respectively, hypophysiotropic Gnrh1, or Gnrh3 in those teleost species lacking Gnrh1, serves as the main stimulator of gonadotropes by projecting in close proximity to gonadotrope cells in the pituitary [for review, see (15)].

Gnrh is regulated by sex steroids in both mammals [for review, see (224) and (225)] and fishes. In teleosts, sex steroids can stimulate or inhibit the activity of Gnrh neurons, thus indirectly regulate gonadotrope function, as shown in **Table 3** and previously discussed in (240). The effects of sex steroids seem to depend on the specific Gnrh cell population and the maturation stage of the fish. For instance, in yearling masu salmon (*Oncorhynchus masou*), castration in under-yearling precocious males increased *gnrh3* mRNA levels in the ventral telencephalon but not in the POA, suggesting that the Gnrh3 cell populations are differentially regulated by gonadal steroids (89). In medaka, E2 significantly suppressed *gnrh* expression in embryos (233) but not in adults (90), suggesting that the effect might be stage-specific in some species. Interestingly, in the spotted scat (*Scatophagus argus)*, E2 inhibited *gnrh1* expression in a dose-dependent manner and this effect was abolished by a broad spectrum Esr antagonist or an Esr1-specific antagonist, but not by an Esr2 antagonist (238), which suggests that Esr1 mediates the inhibitory effect of E2 in this species.

**Table 3:** Effects of sex steroids on the brain and the main neuroendocrine factors involved the regulation of gonadotrope function: Gonadotropin-releasing hormone (Gnrh), Kiss and Dopamine (DA).

Species	Stage	Gnrh	Kiss	DA
Black porgy (*Acanthopagrus schlegelii*)	immature M	E2, no effects on Gnrh1 (51)		
Indian Catfish (*Heteropneustes fossilis*)	pre-spawning F but not in resting phase			E2 ↑ DA (226)
Asian catfish (*Clarias Batrachus*)	juvenile M and F			EE2 ↑ th and DA; MT ↓ th and DA (227)
European eel (*Anguilla anguilla*)	F silver eels	E2 ↑ IRGnrh (228)		
		E2 ↑ Gnrh1; T and Androstenedione ↓ Gnrh2 (229)		
	prepubertal F			T and DHT (not E2) ↑ th (230)
Goldfish (*Carassius auratus*)	adult F		E2 ↑ Kiss2 (191)	
	sexually regressed and recrudescent F			T and E2 ↑ pituitary DA turnover in sexually regressed fish but only T in recrudescent fish (231)
Masu salmon (*Oncorhynchus masou*)	yearling M	MT ↑ Gnrh3 (232)		
	yearling F	MT, no effects on Gnrh3 (232)		
	immature F	MT ↑ Gnrh3 (89)		
Medaka (*Oryzias latipes*)	embryos	E2 ↓ Gnrh expression (233)		
	adult M and F	E2, no effects (90)		
	adult F		E2 ↑ Kiss1 but not Kiss2 (193)	
Orange‐spotted grouper (*Epinephelus coioides*)	adult F		E2 ↓ kiss2 but not kiss1 (92)	
	MT-induced M		MT ↑ *kiss2* (234)	
	immature F	E2 and T ↓ Gnrh (235)	MT ↓ *kiss2* (234)	
Rainbow trout (*Oncorhynchus mykiss*)	immature triploid F	E2 and T ↑ Gnrh3 (102)		
	immature fish	E2, no effect on Gnrh1 or Gnrh2 (236)		
	immature M	T ↑ Gnrh (100)		
	vitellogenic F			E2 ↑ Th (106)
	recrudescent F			E2 ↑ DA and DA metabolites (93)
Sea bass (*Dicentrarchus labrax*)	vitellogenic F	E2 ↓ Gnrh1 (111)	E2, no effects on Kiss1 or Kiss2 (111)	
	mid-vitellogenic F but not in early recrudescence		T, unclear effects on Kiss1 and Kiss2 (237)	
	recrudescent M	T, no effects on Gnrh1 (111)	T ↓ Kiss2 (111)	
Spotted scat (*Scatophagus argus*)	adult F	E2 ↓ Gnrh1 (238)		
Zebrafish (*Danio rerio*)	immature fish		E2 ↑ Kiss1 and Kiss2 (239)	

M, Males; F, Females.

In mammals the limited expression of Esrs in Gnrh neurons indicates that steroids do not exert significant feedback directly to these neurons (for review, see 149 and 150). Similarly, *esr*s were not found in Gnrh neurons in the rainbow trout (241). However, a few studies have reported the presence of Esrs and Ars in teleost Gnrh neurons, suggesting the possibility of a direct sex steroid feedback. The *erα* paralog was found to be expressed in Gnrh3 neurons in medaka (195) and in Gnrh1, 2, and 3 neurons in Nile tilapia (242), while both *arα* and *arβ* were found in Gnrh1 neurons in the cichlid *Astatotilapia burtoni* (179). Thus, it is possible that sex steroids might regulate Gnrh activity and proliferation directly in teleosts, although as described below there is more evidence of indirect pathways *via* effects on Gnrh regulatory factors (e.g., kisspeptin and dopamine), as seen in mammals.

#### Kiss System

Kisspeptin (Kiss), a member of the (RF)-amide peptide family, has been recognized as an important regulator of reproduction in vertebrates. In mammals, Kiss neurons in the POA and the mediobasal hypothalamus are believed to stimulate the synthesis and secretion of Gnrh and mediate feedback by sex steroids (243–245). However, in teleosts, a study in striped bass showed that Kiss regulates gonadotropes in a Gnrh-independent manner (246). Recently, a study in zebrafish reported that Kiss directly stimulates *lhb* and *fshb* expression in pituitary *in vitro* culture, therefore suggesting that Kiss more directly regulates gonadotropes in teleosts than in mammals [(247) and reviewed in (15)].

While *Kiss *expression is stimulated by E2 in rodents (248, 249), the existence of *kiss* paralogs (*kiss1 *and *kiss2*) in teleosts (250, 251) substantially increases the complexity of E2 regulation of *kiss *genes in these species (**Table 3**). Furthermore, which of the paralogs plays a role in the regulation of the BPG axis and steroid feedback may vary by species. For instance, in female medaka, *kiss2* expression in POA does not vary with reproductive state or after OVX (193). However, *kiss1* cell number was higher in reproductive fish compared to that in non-reproductive fish, and decreased significantly after OVX, which suggests sex steroids may exert positive feedback on *kiss1* in this species. In contrast, in the orange-spotted grouper (*Epinephelus coioides*), the expression of *kiss2*, but not *kiss1*, significantly increased in OVX females, which was reversed with E2 treatment (92). However, *in situ* hybridization showed that both *kiss1* and *kiss2* neurons express *esr1*, *esr2a*, and *esr2b*, indicating that E2 may potentially regulate both *kiss1 *and *kiss2 *in this species. Esrs have also been described in kisspeptin neurons in other teleost species: *erα* in medaka (193) and European seabass (217), and *esr1*, *esr2a* and *esr2b* in goldfish (191, 252), suggesting that Kiss neurons may be directly regulated by estrogens in teleosts as in mammals.

Interestingly, androgens also modulate *kiss* expression with different effects depending on the species, sex, or reproductive stage (**Table 3**). For instance, a study in female European seabass reported that during mid-vitellogenesis, but not during early recrudescence, both GDX and T treatment after GDX significantly lowered *kiss1* expression, but that *kiss2* expression decreased only after T treatment in GDX animals (237). In the orange-spotted grouper, during MT-induced sex reversal from female to male, hypothalamic *kiss2* transcript levels were significantly lower 1 week after methyltestosterone (MT) implantation in females (234). Levels remained low in the 2^nd^ and 3^rd^ weeks, but increased significantly in the 4^th^ week, compared to controls. Interestingly, a second MT implant at the 3^rd^ week significantly enhanced *kiss2* expression. These results suggest that MT may stimulate *kiss2* in males but suppress it in females in this species.

#### Dopaminergic System

Dopamine (DA), a catecholamine, is also known to modulate the levels of Gnrh through D2 type DA receptors (253), which subsequently regulate the activity and proliferation of gonadotropes as well as gonadotropin synthesis (17, 254). In teleosts, neurons that produce the tyrosine hydroxylase (Th) enzyme (the rate-limiting enzyme in catecholamine biosynthesis) and project to the pituitary have been localized in the POA, in close proximity of Gnrh neurons, in several species including goldfish (255), rainbow trout (206), European eel (256), zebrafish (257), and the cichlid *A. burtoni* (253).

Several studies have demonstrated the effects of estrogens and androgens on *th* expression or DA levels in teleosts (**Table 3**). The effects of sex steroids on the dopaminergic system seem to also depend on the maturation state. For instance, in the Asian catfish (*Heteropneustes fossilis)*, OVX or E2 replacement in 4-week OVX fish did not significantly affect the DA system during the resting phase, but in the pre-spawning phase, OVX significantly decreased while E2 replacement increased DA levels (226). However, due to the limited number of studies and species investigated, it remains unknown whether the sex steroid regulation of DA is sex- or species-dependent.

Nevertheless, there is evidence that *esr*s are expressed in DA neurons in the POA of rainbow trout brain (206) which suggests a direct role of estrogens on the DA system. However, it is difficult to know whether the observed effects of DA on gonadotropes result from a direct action of DA or if they are indirectly mediated through the Gnrh system, as Gnrh neurons are also controlled by DA.

#### Apoptosis and Proliferation

Sex steroids have been shown to play important roles regulating certain neuronal cell populations in the brain through neurogenesis (29) and neurodegeneration (258) in vertebrates. Published research on the role of sex steroids on cell survival and apoptosis in the teleost brain is limited to a single study in adult male zebrafish, which found that cell survival was slightly reduced in several brain areas after E2 treatment (132).

There is more evidence of the effects of sex steroids on cell proliferation in the teleost brain. For instance, in adult male zebrafish, E2 treatment inhibited cell proliferation in several brain areas, whereas an aromatase inhibitor treatment tended to stimulate cell proliferation, although the effect was not significant in all regions studied (133). However, the same authors reported that fish treated with a high affinity Esr antagonist had higher numbers of proliferative cells in several brain regions, suggesting that E2 inhibits, rather than stimulates, neuronal cell proliferation in this species. An anti-proliferative effect of E2 was also seen in adult female zebrafish, where the number of proliferating cells labelled with BrdU decreased in several neurogenic brain regions, including the POA (214). However, opposite effects were observed in juvenile black porgy treated with E2, where levels of brain aromatase and numbers of proliferative cells increased (259). A significant reduction in brain cell proliferation was observed after treatment with an aromatase inhibitor, while castration did not affect the number of brain cells. This suggests that T itself may inhibit neurogenesis, but local E2 synthesis from aromatization of T may promote neurogenesis in this species.

Changes in specific neuroendocrine cell populations in teleosts due to sex steroids was first investigated in sex‐reversing fishes. In bluehead wrasse (*Thalassoma bifasciatum*), the number of Gnrh neurons in the POA was higher in males at the terminal phase of sex transformation than females or initial-phase males (260, 261). Additionally, the number of Gnrh neurons was shown to increase in females and initial-phase males, but not in terminal phase males, following 11-KT treatment, demonstrating a role of sex steroids on Gnrh neuron number (260, 261). However, whether this increase was due to cell proliferation or recruitment was not investigated. In Mozambique tilapia, Gnrh3 neurons are more numerous in males, and treatment with 11-KT or methyltestosterone (MT, a potent synthetic androgen), but not E2, increased the number of Gnrh3 neurons in females to a level similar to that in males, and modified the fish behavior (262). Recently, the same group showed that this phenomenon was due to proliferation by identifying newly formed Gnrh3 neurons after androgen treatment (263). In larval zebrafish, treatment with the synthetic estrogen 17α-ethinylestradiol (EE2) increased the numbers of forebrain Gnrh3 cells (264). The authors suggest that EE2 accelerated Gnrh3 neuron development as 5 dpf larvae treated with EE2 had similar numbers of Gnrh3 neurons as 20 dpf control fish. Similar effects of sex steroids were seen in immature African catfish, where testosterone increased the number of Gnrh1 neurons (265).

There is also some evidence that sex steroids may also affect kiss neuron number in teleosts. In medaka, the number of *kiss1* neurons was observed to decrease after OVX in some brain regions, but was maintained with E2 treatment (193). However, it is not known whether this was due to a decrease of *kiss1* expression in some neurons after OVX or if E2 treatment had a positive effect on cell survival. Finally, information on the effects of sex steroids on DA cell number is to date still lacking.

### Direct Effects on Gonadotropes

Because no direct effects of sex steroids have been demonstrated on gonadotrope proliferation, transdifferentiation or cell death *in vitro*, this section will address the question of gonadotrope plasticity by considering changes in their activity only.

#### The Pituitary: A Target of Sex Steroids

As in other vertebrates, Esrs have been identified in the pituitary of many teleost species while Ars have been described in only a few species (**Table 2**).

Interestingly, in male wild sablefish (*Anoplopoma fimbria*), pituitary *esr1* and *ara* mRNA levels were positively correlated with those of *lhb*, whereas *esr2a* and *esr2b* were correlated with *fshb* transcripts during gametogenesis (213). However, whether the alterations in *esr* or *ar* levels occurred in gonadotropes remains to be determined. Indeed, the specific cell types within the pituitary that express these receptors have only been investigated in a few teleost species. In European sea bass, high expression of *esr1*, *esr2a*, and *esr2b* mRNAs was localized to the PPD and PI, and double label *in situ* hybridization demonstrated that both Fsh and Lh cells express *esr1*, *esr2a*, and *esr2b* transcripts (215, 216). In ricefield eel (*Monopterus albus*), *esr1*, but not *esr2*, was expressed in Lh cells (211, 212). In medaka, Lh cells expressed all three isoforms, with *esr1* and *esr2b* most predominately expressed (91). In contrast, nothing is known regarding the presence of *ar*s in teleost gonadotropes.

In summary, the localization of both Esrs and Ars in the pituitary, and the presence of Esr in gonadotropes support a direct effect of sex steroids on gonadotropes. Yet, further studies are needed to identify which pituitary cell types express which receptors and thereby provide stronger evidence of direct signaling, and to determine whether expression patterns vary among species and with stage of sexual maturity.

#### Aromatization of Androgens in the Pituitary: A Production Site of Estrogens

Using aromatase inhibitors (51, 211, 266) or a combination of aromatizable and non-aromatizable androgens (62, 79, 91, 116), several studies have clearly demonstrated that aromatase, by converting T into E2, plays a role in the cellular responses to T observed in teleosts.

Aromatase has been identified in the pituitary of all major vertebrate groups from fishes to mammals. However, experiments in goldfish, toadfish (*Opsanus tau*), and sculpin (*Myoxocephalus octadecimspinosus*) showed that teleost pituitaries have aromatase levels l00 –1000 times greater than those in mammals and other vertebrates (27, 267, 268). Since then, aromatase expression or activity has been demonstrated in the pituitary of many other teleost species such as African catfish (269), Atlantic salmon (270), Mozambique tilapia (*Oreochromis mossambicus)* (271), rainbow trout (210), channel catfish (*Ictalurus punctatus*), and zebrafish (272), midshipman fish (*Porichthys notatus*) (196), Atlantic cod (273), killifish (*Fundulus heteroclitus*) (274), black sea bass (*Centropristis striata*) (275), a neotropical cichlid fish (*Cichlasoma dimerus*) (276), sablefish (213), brown ghost knifefish [*Apteronotus leptorhynchus*) (277)], and black porgy (51).

Interestingly, in pejerrey (*Odontesthes bonariensis*), aromatase (*cyp19a1b*) expressing cells labeled by immunohistochemistry or *in situ* hybridization were found close to blood vessels in the pituitary (278). Moreover, aromatase has been located in pituitary cells in the Japanese eel (152) and larval zebrafish (264), in Lh cells, but not Fsh cells, in ricefield eels (211), and in both Lh and Fsh cells in medaka (91, 116). While no sex differences were detected in pituitary aromatase mRNA levels or enzyme activity in the pejerrey (278) and the Japanese eel (279), respectively, aromatase activity in European seabass (280), and *cyp19a1b* expression in the yellow perch (281) and a South American catfish (282), were higher in male pituitaries. This might explain the differential sex responses of gonadotropes observed following steroid treatment in some species. In larval zebrafish, EE2 exposure did not affect *cyp19a1b* levels in the pituitary, despite increasing levels in the forebrain (264).

#### Direct Effects on Hormone Synthesis and Release

In 1983, pituitary grafts (transplantation of pituitaries to another tissue) in rainbow trout revealed positive effects of T on pituitary and plasma Lh levels, indicating a stimulatory role of T on Lh synthesis and release (283). Stimulatory effects of androgens and estrogens have also been observed in studies using whole pituitary or fragments in cultures from rainbow trout (99) and ricefield eel (211). However, as paracrine signaling occurs between pituitary cells in vertebrates (284), including in teleosts as shown for instance in goldfish (285) and Grass carp (*Ctenopharyngodon idella*) (286), and pituitary endocrine cells can be stimulated by neuroendocrine factors despite the absence of cognate receptors, through cell-cell communication (176), using whole pituitary or fragments could mask potential direct effects from the steroids. Therefore, we argue that dissociated cell culture is the only suitable technique to investigate the direct effect of sex steroids (or other factors) on gonadotropin synthesis and secretion.

Studies using dissociated pituitary cell cultures from several teleost species have yielded convincing evidence for a direct role of sex steroids in the regulation of gonadotropes, mostly by stimulating *lhb* and *fshb* expression (**Table 4**). However, divergent effects of treatment duration have reported in juvenile eel pituitary cells for example, where no effects on *lhb* mRNA levels were observed after 24 h E2 treatment while a 72 h E2 treatment decreased it (288). In African catfish, both T and E2 inhibited *lhb* transcription after 24 h treatment but simulated *lhb* levels after 48 h treatment (287).

**Table 4:** Direct effects of sex steroids demonstrated by *in vitro* studies using dissociated pituitary cells from teleosts.

Species	Stages	Synthesis		Secretion	References
		Lh	Fsh	Lh	
African catfish (*Clarias gariepinus*)	mature M	24 h treatment E2 and T ↓ *lhb* 48 h treatment: T and E2 ↑ *lhb* (not DHT)			(287)
Atlantic cod (*Gadus morhua*)	maturing mix F/M	No effect of T, DHT, or E2 on *lhb*	T ↑ *fshb* (not E2 or DHT)		(52)
	mature mix F/M	DHT ↑ *lhb* (not T or E2)	E2 DHT ↑ *fshb* (not T)		
	Post-spawning mix F/M	No effect of T, DHT, or E2 on *lhb*	T ↓ *fshb* (not E2 or DHT)	
Black porgy (*Acanthopagrus schlegelii*)	mature M			no effects of E2, T, 11-KT on basal levels 11-KT and E2 ↑ Gnrh-induce stimulation	(69)
Channel catfish (*Ictalurus punctatus*)	mature F	E2 and T ↑ *lhb*			(266)
European eel (*Anguilla anguilla*)	silver F	T, DHT and 3α-diol ↑ *lhb* and Lh 24 h treatment: no effect of E2 on *lhb* or Lh 72 h treatment: E2 ↓ *lhb*		T, DHT and 3α-diol ↑ (not E2)	(288)
		T ↑ *lhb* and Lh (not E2)			(289)
		T and DHT ↑ *lhb* (not E2)	E2 ↑ *fshb* (not T or DHT)		(77)
Goldfish (*Carassius auratus*)	mature or sexually regressed	no effect of T on *lhb*	no effect of T on *fshb*		(290)
	immature	*T ↑ lhb*	no effect of T on *fshb*	
Hybrid tilapia (*Oreochromis niloticus × O. aureus)*	immature M	No effect of T on *lhb*	*T ↑ fshb*		(291)
Marine medaka	mature mix M/F	E2 ↑ *lhb*	E2 ↑ *fshb*		(292)
Masu salmon (*Oncorhynchus masou*)	F and M	E2 and T ↑ *lhb* (not 11-KT)		E2 and T ↑ (not 11-KT)	(293)
		E2 ↑ *lhb* and Lh	no effect of E2 on *fshb* or Fsh		(294)
Nile tilapia (*Oreochromis niloticus*)	Maturing F	E2 ↑ *lhb*	no effect of E2 on *fshb*		(49)
Rainbow trout (*Oncorhynchus mykiss*)	immature	T ↑ Lh		T ↑	(283)
	Adult F			E2 ↑ Gnrh-induce stimulation	(295)
	Immature F			E2 ↑ Gnrh-induce stimulation	(57)
Ricefield eel (*Monopterus albus*)	??	E2 ↑ *lhb*	no effect of E2 on *fshb*		(212)
Zebrafish (*Danio rerio*)	mature mix F/M	T and E2 ↑ *lhb*	T and E2 ↑ *fshb*		(296)

Interestingly, in the channel catfish, both E2 and T enhanced the expression of *lhb*, but the effect of T was abolished by an aromatase inhibitor (266), indicating an important role of aromatase and E2. This result is supported by other studies where T, but not non-aromatizable androgens, gave a similar effect on gonadotropin gene expression as did E2 treatment (77, 293). Several studies using dissociated pituitary cell cultures have also demonstrated a direct effect of sex steroids on Gnrh-induced Lh release (**Table 4**). For instance, E2 treatment increased Gnrh3-stimulated Lh release from female rainbow trout pituitary cells (57, 295).

Again, sex steroid effects on gonadotropes were found to vary with stage of sexual maturity and sex. For example, T stimulated *fshb* in cells from maturing Atlantic cod, had no effect in cells from mature fish and decreased *fshb* from post-spawning fish (52). In cells from masu salmon, the combination of Gnrh3 and E2 increased *lhb* mRNA levels and decreased those of *fshb* in males, but had no effect on *lhb* or *fshb* in females (297). These results indicate that E2 and Gnrh3 signaling differentially modulate gonadotropin synthesis and that effects might be sex-specific in this species.

Further evidence of the ability of sex steroids to directly modulate gonadotropin transcription is provided by the identification of steroid response elements (SRE) in gonadotropin promoters and by *in vitro* reporter assays. SRE are short, palindromic nucleotide sequences in target genes where steroid receptors bind to regulate transcription of those genes. Full-site and half-site estrogen response elements (ERE) have been identified upstream of the *lhb* gene in chinook salmon (298, 299), but only half-site EREs have been found in both *fshb* and *lhb* genes in Nile tilapia [*lhb* (300); *fshb* (301)] and goldfish [*lhb* (80); *fshb* (302)], and the *fshb* gene in chinook salmon (303) and sea bass (304). A half-site androgen response element (ARE) was also been identified upstream of *fhb* in sea bass (304). Using an *in vitro* reporter assay, the ricefield eel *lhb* promoter was activated highly by E2, and to a lesser extent by T and 11-KT, indicating the presence of functionally active ERE and ARE in the *lhb* promoter. Conversely, neither E2 or androgens activated the *fshb* promoter (211).

### Conclusion

As shown above, it is clear that sex steroids act at both the brain and gonadotrope levels. However, there is still a need to further decipher direct from indirect effects of sex steroids. So far, the use of dispersed cell cultures has been the only way to confirm a direct effect of sex steroids on gonadotropes. However, precaution should be taken as gonadotropes have been shown to change phenotype after dissociation and seeding. Indeed, in a medaka study, Fsh cells which were shown to not possess Gnrh receptors *in vivo* and to not respond to Gnrh treatment *in vitro* 24 h after dissociation, did respond to Gnrh after 3 days in culture (116), suggesting that they begin to express Gnrh receptors during incubation. Therefore, the direct effects of sex steroids in dissociated cell cultures should be confirmed by the localization of sex steroid receptors *in vivo* in the cells of interest.

A lot of work remains to identify the cell types that express *esr* and *ar*, in both the brain and the pituitary which will help to elucidate the regulatory pathway. While Gnrh, Kiss and DA are the primary gonadotrope regulators, they are complemented by, and themselves controlled by, other factors including sex steroids. New techniques such as multicolor *in situ* hybridization and single cell transcriptomics will accelerate such research.

Finally, most of the direct effects observed on gonadotrope plasticity are about the activity (cell sensitivity and hormone synthesis and release). No data exist on the direct effect of sex steroids on the regulation of cell proliferation or cell death, which are difficult to address in *in vitro* work. Therefore, new approaches are needed to address these questions in the future.

## General Conclusion and Future Perspectives

To conclude, any attempt to draw generalizations in teleosts, or to propose hypotheses on evolutionarily conserved mechanisms or ecological, developmental or sex-specific adaptations, is difficult due to an insufficient body of evidence. Indeed, although many studies have been performed on several species, the information is often conflicting, with differential effects of sex steroids among species, sexes, developmental stages, and reproductive states. Because effects can vary between males and females, or over the reproductive cycle within one species, it is evident that the signaling pathways can change according to the unique molecular environment. It must be recognized that teleost fish diverged over their 300 MY evolutionary history, and there is enormous variability among its nearly 30,000 species, in terms of both the environmental parameters of their habitats (salinity, light, temperature, etc.) and their reproductive strategies (iteroparity vs. semelparity, daily spawning vs. seasonal spawning, etc.). Thus, it is not surprising to find diversity among teleosts regarding the roles of sex steroids in mediating gonadotrope plasticity and the pathways by which they exert these effects. This high complexity of sex steroid signaling necessitates further research addressing the molecular mechanisms behind the observed responses. One mechanism underlying such variation is likely the differences in steroid levels across stages and between sexes, which has been extensively reviewed [e.g. (18, 305–307)]. A second mechanism is likely due to variation in the steroid modifying enzymes (e.g., aromatase) in the pituitary, leading to local changes in levels of specific steroids. A third mechanism is likely differences in the receptor subtypes and their numbers in target tissues, which might be differentially regulated by environmental and internal factors.

Fortunately, we have a wealth of powerful tools available to investigate the role of gonadal sex steroids. For instance, GDX, which is still used today in teleosts and adapted to new species of interest (308), is a powerful technique when combined with steroid replacement to investigate the role sex steroids play in tissue plasticity. However, GDX does not remove all sex steroids, as some can be produced by interrenal cells and adipose tissue (309), as well as in the brain and pituitary as described above. Therefore, future studies are needed to investigate the potential roles of the extra-gonadal sources of sex steroids. Administration with exogenous hormones is certainly an appropriate approach to evaluate physiological effects, but the method of administration, time and duration, and concentration can influence response. For example, recent studies in medaka showed that E2 may be best administered through feeding as it is convenient and effectively mimics the diurnal E2 changes in this species, whereas fish exposed to E2 in tank water exhibited blood E2 concentrations exceeding those of environmental water, suggesting that E2 bioconcentrates (310). In addition, as shown in the female ricefield eel for example (211), when testing for effects of steroids *in vivo*, the use of a non-aromatizable androgen such as 11-KT and an aromatase inhibitor in addition to androgen and estrogen treatments will help to clearly identify the roles of estrogens vs. androgens, which is still unclear.

Finally, because teleosts possess many paralogous sex steroid receptors, it is important that future studies investigate the role of each. Recently, transgenesis techniques such as TALEN and CRISPR/Cas9 have provided new approaches for such investigations. In medaka, for example, TALEN was used to develop an Esr1 knockout (KO) fish (311). Using these animals, the authors demonstrated the dispensable role of Esr1 for development and reproduction in medaka. Using the CRISPR/Cas9 technique, three mutant transgenic zebrafish lines have been created for each of the *esr* present in the zebrafish genome, as well as all possible double and triple knockouts of the three *esr*s (312). The authors did not observe any reproductive dysfunction for the three single *esr* mutant fish lines, which suggests functional redundancy among Esrs. However, double and triple knockouts showed that *esr2a* and *esr2b* were essential for reproduction in females and maintenance of the female sex phenotype as the double mutant sex-change from female to male. While these techniques show a great number of benefits, some limitations still exist. Knocking out gene expression from the one cell stage, as it is currently performed in most teleost experiments, may activate compensatory mechanisms (312). Also, as sex steroid receptors, aromatase, and other steroidogenic enzymes are widely expressed in the brain, pituitary and gonads, it is impossible to identify the precise origin of the effects observed after a KO is made. However, techniques allowing spatial and/or temporal control of the KO have recently been established in fish (313). Such techniques might be a promising tool for future investigations of the molecular, cellular and physiological roles of specific sex steroid receptors, and thus their roles in gonadotrope plasticity.

Thus, we are hopeful that more light will be shed on these topics as they will provide important information to better understand the role of sex steroids and the pathway they use to regulate gonadotrope plasticity.

## Author Contributions

RF, DB, and F-AW planned the manuscript. RF and DB wrote the paper with the help of MR and KK. All authors contributed to the article and approved the submitted version.

## Funding

This study was funded by the Norwegian University of Life Sciences. DB was supported by the Waple Professorship at the University of Mary Washington.

## Conflict of Interest

The authors declare that the research was conducted in the absence of any commercial or financial relationships that could be construed as a potential conflict of interest.

## Abbreviations

11- HA, 11β-hydroxyandrosterone; 11-KT, 11-ketotestosterone; Acth, Adrenocorticotropin; Ar, Androgen receptor; BPG, Brain-pituitary-gonad; D1, Type 1 dopamine receptors; D2, Type 2 dopamine receptors; DA, Dopamine; DHT, 5α-dihydrotestosterone; E2, 17β-estradiol; EE2, 17α-ethinylestradiol; Esr, Estrogen receptor; Fsh, Follicle-stimulating hormone; GDX, Gonadectomy; Gh, Growth hormone; Gnrh, Gonadotropin-releasing hormone; Lh, Luteinizing hormone; Msh, Melanocyte-stimulating hormone; MT, Methyltestosterone; OVX, Ovariectomy; PI, Pars intermedia; POA, Preoptic area; Pomc, Pro-opiomelanocortin; PPD, Proximal pars distalis; Prl, Prolactin; RPD, Rostral pars distalis; Sl, Somatolactin; T, Testosterone; Th, Tyrosine hydroxylase; Tsh, Thyrotropin.
